# Desmosterol and 7-dehydrocholesterol concentrations in post mortem brains of depressed people: The role of trazodone

**DOI:** 10.1038/s41398-022-01903-3

**Published:** 2022-04-04

**Authors:** Basar Cenik, Jayme M. Palka, Bonne M. Thompson, Jeffrey G. McDonald, Carol A. Tamminga, Can Cenik, E. Sherwood Brown

**Affiliations:** 1 SIU Neuroscience Institute, Department of Psychiatry, Southern Illinois University School of Medicine, 319 E Madison Street, Springfield, IL 62794-9642 USA; 2 Department of Psychiatry, The University of Texas Southwestern Medical Center, 5323 Harry Hines Blvd. MC 8849, Dallas, TX 75390-8849 USA; 3 Center for Human Nutrition, The University of Texas Southwestern Medical Center, 5323 Harry Hines Blvd. MC 8849, Dallas, TX 75390-8849 USA; 4 Department of Molecular Genetics, The University of Texas Southwestern Medical Center, 5323 Harry Hines Blvd. MC 8849, Dallas, TX 75390-8849 USA; 5 Department of Molecular Biosciences, University of Texas at Austin, 2500 Speedway, Austin, TX 78705 USA

**Keywords:** Molecular neuroscience, Predictive markers, Depression

## Abstract

Major depressive disorder (MDD) is a common, disabling, and heterogeneous condition that responds unpredictably to current treatments. We previously showed an association between depressive symptoms and plasma concentrations of two cholesterol precursors, desmosterol and 7-dehydrocholesterol (7DHC). Here, we measured total cholesterol and sterol concentrations with mass spectrometry in postmortem brain samples from depressed and control subjects. Mean (±SEM) desmosterol concentration was 8.9 ± 0.97 ng/mg in the depressed versus 10.7 ± 0.72 ng/mg in the control group. The mean of the posterior probability distribution for the difference in desmosterol concentration between the two groups was 2.36 (95% highest density interval [HDI] 0.59–4.17). Mean 7DHC concentrations, 12.5 ± 4.1 ng/mg in the depressed versus 5.4 ± 0.74 ng/mg in the control group, were unlikely to be different (95% HDI, [−1.37–0.34]). We found that presence of trazodone in the peri-mortem toxicology screen accounted for the observed difference in desmosterol concentrations. We also observed extremely high 7DHC levels in all 4 subjects who had taken trazodone. Trazodone has been recently found to inhibit 7-dehydrocholesterol reductase and alter sterol concentrations in rodents, cell culture, human fibroblasts, and blood. In this study, we demonstrate for the first time that trazodone alters human brain sterol composition. Given congenital deficiency of 7-dehydrocholesterol reductase results in Smith-Lemli-Opitz syndrome, our findings support the hypothesis that this commonly used medication may have previously unappreciated risks.

## Introduction

Major depressive disorder (MDD) is one of the top two causes of disability in the world [1–3]. Lifetime prevalence in North America is estimated to be ~15% [4]. Current treatments are only partially effective; about one third of patients with MDD fail to achieve remission with first or second line treatments [5]. One hypothesis that potentially explains the unpredictable response to treatment is the biological heterogeneity of the diagnostic construct [6, 7]. A promising and topical approach to this problem is the discovery and validation of depression biomarkers.

Cholesterol is essential for brain function [8]. Additionally, a putative association between low plasma cholesterol and depression or suicide has been suggested by several lines of investigation [9–14]. In light of these findings, we previously investigated associations between plasma sterols and depressive symptoms in a large, population-based cohort, as potential biomarkers [15]. We found that lower concentrations of the cholesterol precursor desmosterol and higher concentrations of another precursor, 7-dehydrocholesterol (7DHC), were predictive of moderate to severe depressive symptoms.

Since the publication of our study, it has become increasingly apparent that several psychotropic medications interfere with cholesterol synthesis [16–21]. In the current study, we tested the hypothesis that desmosterol and 7DHC would be altered in postmortem brains from people with depression and these changes may be explained by psychotropic medication use.

This is, to our knowledge, the first study to investigate cholesterol precursors and other related sterols in a comprehensive manner in postmortem brains. We focused on two brain regions: the prefrontal cortex (PFC) and the cerebellum. PFC was chosen given the well-known implications in the neurobiology of MDD [22–27] and as the main target of emerging neuro-stimulation treatments. Cerebellum has also been implicated as a part of brain networks disrupted in MDD [28–30] but is anatomically distinct from the PFC.

## Materials, subjects and methods

### Study population

Frozen, pulverized postmortem human brain samples and de-identified clinical data were obtained from Dallas Brain Collection. The prefrontal cortex (PFC) samples were from Brodmann area 9 in the dorsolateral PFC. Cerebellar (CBL) samples were dissected from the cerebellar cortex, avoiding the vermis. MDD diagnoses were made using all available information, including review of medical records and phone calls to caregivers when necessary. These data were presented to at least two (usually 3–4) experienced clinicians and diagnoses were made according to Diagnostic and Statistical Manual criteria. Samples from subjects diagnosed with MDD were matched to samples from subjects with no diagnosis of depression based on age and postmortem interval. Medication use data was obtained from medical charts and also from peri-mortem toxicology reports. Subject characteristics are summarized in Table 1. Sample size was estimated based on the effect size we had observed in plasma samples in a previous study [15]. Collection of postmortem brains by the Dallas Brain Collection was approved by UT Southwestern Institutional Review Board. The investigators were blinded to sample group assignments during mass spectrometry and initial analysis steps. The Dallas Brain Collection kept the master file with sample group assignments. The blind was broken after primary analyses were completed and some secondary analyses done un-blinded.

**Table 1:** Subject characteristics.

	Depressed (*n* = 20)	Control (*n* = 20)
Age (mean, range)	51.8 (17–77)	51.6 (19–74)
Female (%)	40	25
Caucasian (%)	95	85
Postmortem interval in hours (mean, range)	14.8 (7.4–28)	17.3 (8.3–24)
RNA integrity number (mean, range)	7.52 (6.03–9.90)	8.36 (5.90–10.00)

### Sterol concentration measurements from postmortem brain tissue

Samples were processed and analyzed as described in McDonald et al. [31]. Briefly, samples were rapidly thawed and hydrolyzed to generate a pool of free sterols, extracted with a modified Bligh/Dyer 2-phase extraction, and purified with solid-phase extraction. Sterols were analyzed using LC-MS as previously described [31]. In total, we measured concentrations of 19 sterols, including total cholesterol.

### Statistical analyses

To examine whether healthy controls and depressed subjects differ on their mean sterol levels, independent samples t-tests with robust Bayesian estimation were conducted using the BEST package [32] in R version 3.6.3. Differences in mean sterol concentrations were tested for 7DHC and desmosterol using brain tissues from the prefrontal cortex (PFC) and cerebellum (CBL). As opposed to a frequentist t-test, the robust Bayesian estimation can accommodate the presence of outliers. Model results reveal the relative credibility for each possible parameter value in the form of a posterior probability distribution. The 95% highest density interval (HDI) was calculated using the default MCMC parameters with 1000 burn-in steps. Convergence was reached for all parameters as measured by pre-defined criteria of Brooks-Gelman-Rubin scale reduction factor <1.1.

In a Bayesian framework, hypothesis testing is comparative and the likelihood of observed data is considered under both the null and alternative. In contrast, frequentist statistics do not explicitly calculate the probabilities of the alternative hypothesis and numerous drawbacks of using frequentist statistics have been extensively discussed in the literature [33, 34].

The choice to use a Bayesian framework for the present study was two-fold. First, prior work has shown that blood sterol concentrations are significantly related to depressive symptoms [15]. The use of a Bayesian method enables incorporation of prior knowledge in our present analysis. Second, Bayesian estimation allows one to express the level of uncertainty in the results using a 95% credibility interval (CI) around the most likely parameter values.

## Results and discussion

**Fig. 1:**
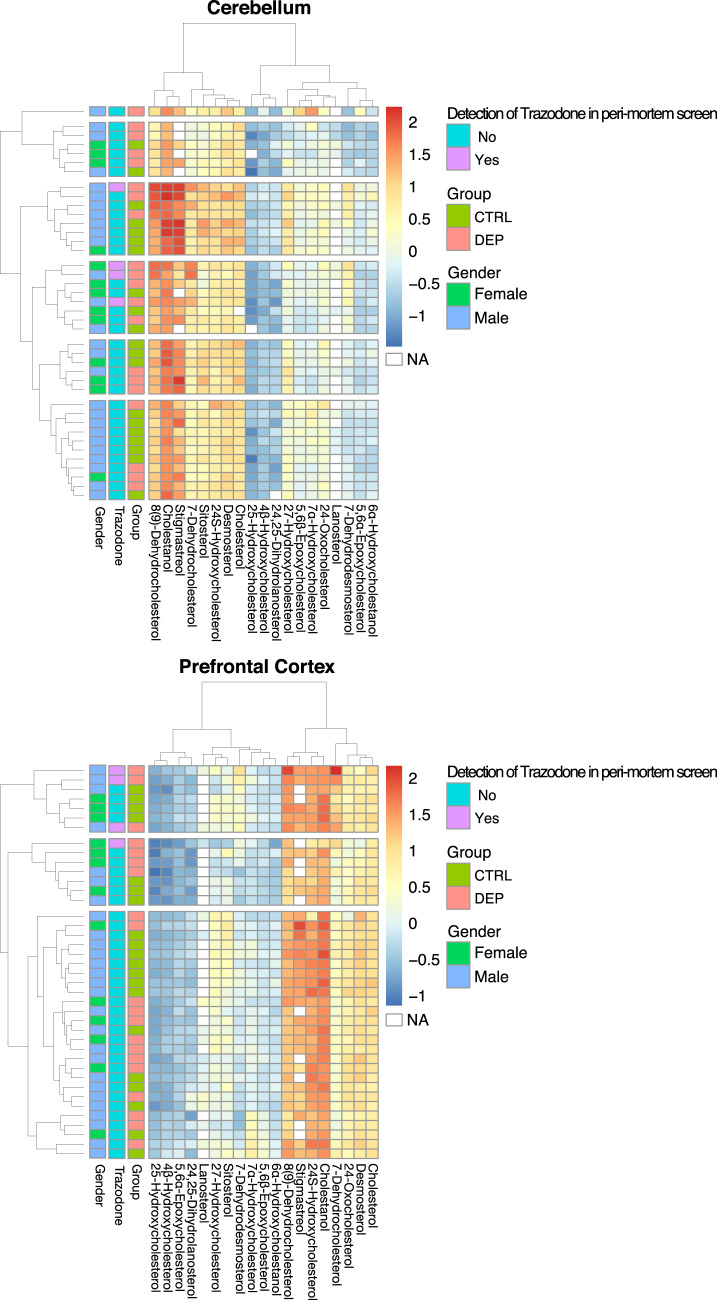
Sterol concentrations in post-mortem brain tissue samples. The log_10_ of sterol concentrations for each individual is shown with the color scale. The samples were clustered and visualized using pheatmap package in R. CTRL control; DEP depressed.

Demographic variables of subjects and postmortem tissue quality markers are summarized in Table 1. Controls were age matched to depressed samples. Tissue quality markers were similar in both groups. Concentrations of sterols were measured in PFC and cerebellum samples from 20 depressed subjects and 20 controls (80 samples in total). Total cholesterol was also measured in 79 of the samples (one cerebellum sample from the control group did not have enough material for this measurement). Concentrations of 19 sterols in depressed and control samples are summarized in Fig. 1. There was no significant association between gender and sterol levels.

**Fig. 2:**
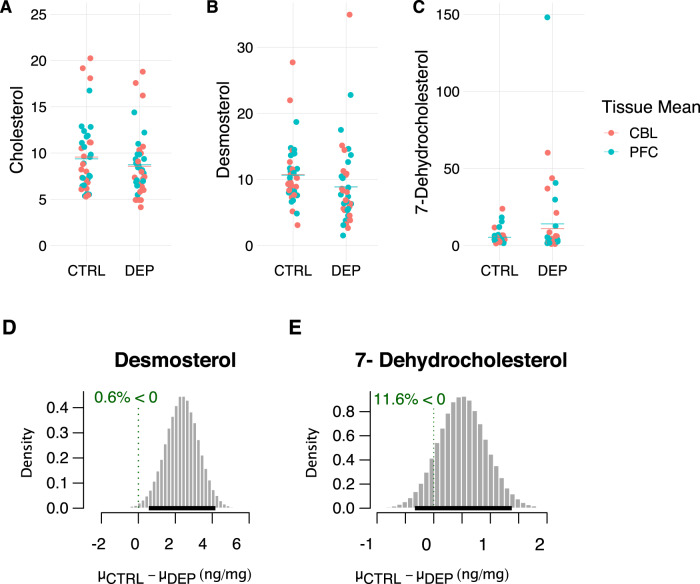
Difference in mean sterol concentrations between control and depressed groups. **A** Cholesterol (μg per mg tissue), (**B**) desmosterol (ng per mg tissue), and (**C**) 7-dehydrocholesterol (ng per mg tissue) measurements were made in two brain regions: cerebellum (CBL) and pre-frontal cortex (PFC), from control (CTRL) and depressed (DEP) subjects. The thick lines correspond to the group and brain region specific mean sterol concentrations. **D** Posterior probability distribution of the difference in mean desmosterol concentrations between control (μ_ctrl_) and depressed (μ_dep_) groups are plotted; the values are in ng/mg tissue. The mean of the distribution is 2.4 ng/mg. Dark horizontal line above the axis indicates the 95% Highest Density Interval (0.59–4.17 ng/mg). Green dotted line indicates the probability that the difference in the means is less than or equal to zero (0.006). **E** Posterior probability distribution of the difference in mean 7DHC concentrations between control and depressed groups is plotted as in (A). The mean of the distribution is 0.52 ng/mg (95% Highest Density Interval −0.34–1.38 ng/mg). The probability that the difference in the means is less than or equal to zero is 0.116.

Total cholesterol abundance was nominally lower in the depressed group both in the PFC and the cerebellum but unlikely to be different (95% highest density interval [HDI] of mean difference, −0.75–2.24 μg/mg) (Fig. 2A; Table 2). Measurements from PFC and cerebellum samples had similar mean values for cholesterol, desmosterol, and 7DHC in both groups (Fig. 2B, C; Table 2). Incidentally, we found lower 24S-hydroxycholesterol concentrations in the cerebellum (Fig. S4). Desmosterol and 7DHC abundance correlated weakly at the level of individual subjects between the two brain regions (Fig. S1; biweight midcorrelation coefficient 0.19 and 0.35, respectively). Therefore, we pooled PFC and cerebellum measurements for further analysis.

**Table 2:** Sterol concentrations.

	Depressed	Control
Total Cholesterol (μg/mg tissue, mean ± SEM)		
Cerebellum	8.6 ± 1	9.6 ± 1
PFC	8.8 ± 0.5	9.4 ± 0.7
Desmosterol (ng/mg tissue, mean ± SEM)		
Cerebellum	8.9 ± 1.6	10.7 ± 1.2
PFC	8.9 ± 1.2	10.7 ± 0.8
7DHC (ng/mg tissue, mean ± SEM)		
Cerebellum	10.9 ± 3.7	5.4 ± 1.1
PFC	14.1 ± 7.4	5.5 ± 1

*SEM* standard error of the mean, *PFC* prefrontal cortex, *7DHC* 7-dehydrocholesterol.

We hypothesized that desmosterol concentrations would be lower in depressed samples compared to controls based on our previous finding of lower plasma desmosterol levels in depressed subjects in an independent sample. Here, we found a mean (±SEM) desmosterol concentration of 8.9 ± 0.97 ng/mg in the depressed versus 10.7 ± 0.72 ng/mg in the control group (Table 2). We used an independent samples t-test with robust Bayesian estimation and found that the mean of the posterior probability distribution for the difference in desmosterol concentration between the two groups was 2.36 ng/mg (95% highest density interval [HDI] 0.59–4.17 ng/mg) (Fig. 2D). The probability that the difference in the means is less than or equal to zero was 0.006 (Fig. 2D). Sensitivity analyses using only PFC or only cerebellum measurements revealed similar results (Fig. S2). We also found the six highest brain 7DHC levels (from 4 individual subjects) in the depressed group (Fig. 2C). This results in a higher mean 7DHC concentration in the depressed group with a large variance (12.5 ± 4.1 ng/mg in the depressed versus 5.4 ± 0.74 ng/mg in the control group; Table 2). However, using independent samples t-tests with robust Bayesian estimation we found that the mean 7DHC concentrations between the two groups (depressed minus control) is unlikely to be different (95% HDI, [−1.37–0.34]) (Figs. 2E; S3).

**Fig. 3:**
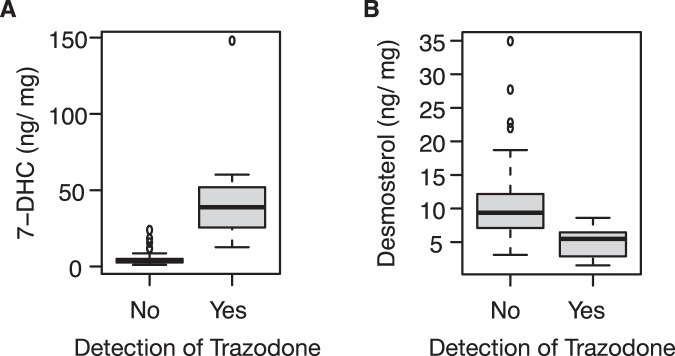
Post-mortem brain sterol concentrations in individuals as function of trazodone use. Subjects are separated into two groups based on detection of trazodone in a peri-mortem toxicology screen. The distribution of (**A**) 7-dehydrocholesterol (7-DHC) and (**B**) desmosterol is depicted as a boxplot. The median is indicated with the thick horizontal black line and the box correspond to the interquartile range. Individual measurements that are outside 1.5 times the interquartile range are depicted with separate dots.

Next, we investigated whether the differences in brain sterol concentrations could be explained by medication use, based on recent reports of inhibition of key cholesterol synthesis enzymes by psychotropic medications [17–19, 35, 36]. We considered data from both medication use history and toxicology reports, and identified morphine, ethanol, trazodone, escitalopram, alprazolam, diphenhydramine, hydrocodone, aspirin, atropine, insulin, and lisinopril as the only medications used by or found in >2 subjects. Only trazodone was significantly correlated with low desmosterol and high 7DHC concentrations (Spearman rho −0.38, and 0.51 with corresponding *p* values of 0.0005 and 0.0000012, respectively). As shown in Fig. 3, trazodone use explains all the difference in desmosterol and 7DHC levels between the groups. Specifically, all 4 subjects with extremely high 7DHC levels had taken trazodone; desmosterol levels in depressed subjects who did not take trazodone were indistinguishable from levels in the control group (Wilcoxon rank sum test *p* = 0.122); depressed subjects who took trazodone had significantly lower desmosterol levels compared to others (Wilcoxon rank sum test *p* = 0.009). Last, we found trazodone use also correlated with increased 7-dehydrodesmosterol and 8(9)-dehydrocholesterol concentrations (Spearman rho 0.48, and 0.43 with corresponding *p* values of 0.0000063 and 0.00007, respectively). Taken together, trazodone alters brain sterol composition.

Our prior work has shown that plasma levels of two cholesterol precursors, desmosterol and 7DHC, can be used to predict depressive symptoms in a population. This finding suggested plasma sterol levels may be depression biomarkers. One of the major limitations of our previous study was that the correlation between plasma and brain levels of these sterols was unknown. Additionally, we were not able to analyze potential confounding by medication use. The current study addresses both limitations. Here, we show that desmosterol and 7DHC abundances have the same direction of difference in brains from clinically depressed subjects as they had in the plasma of subjects with moderate to severe depressive symptoms in a different cohort. However, this difference seems to be wholly explained by the use of trazodone, a sedating antidepressant.

Cholesterol is indispensable for brain development and function [37]. Changes in brain cholesterol concentrations have long been associated with suicide and depression. More recently, four psychotropic medications, aripiprazole, cariprazine, haloperidol, and trazodone have been shown to be inhibitors of 7-dehydrocholesterol reductase, leading to increased 7DHC and reduced desmosterol levels in cell culture, rodents (including in utero models), human dermal fibroblasts, and human blood [17, 19, 20, 35, 36, 38]. In these models, the magnitude of 7-DHC elevation was comparable to levels seen in Smith-Lemli-Opitz syndrome, a severe congenital syndrome caused by mutations in the DHCR7 gene encoding 7-dehydrocholesterol reductase. Recent reviews have raised caution about the use of these medications in pregnancy and in carriers of DHCR7 mutations [17]. Our study, to the best of our knowledge, is the first demonstration of sterol abnormalities in human brains due to trazodone use. This is a previously unappreciated potential risk of this commonly used medication. The current package label for trazodone (Desyrel^®^) (https://www.accessdata.fda.gov/drugsatfda_docs/label/2015/071196s062lbl.pdf) contains sparse information about pregnancy implications and no warnings about potential effects on cholesterol synthesis *in utero*, highlighting the need for increased pharmacovigilance in this area.

## Supplementary information

Supplementary figure legends

Figure S1

Figure S2

Figure S3

Figure S4
