# Supplementary material for: Desmosterol and 7-dehydrocholesterol concentrations in post mortem brains of depressed people: The role of trazodone
Source: Transl Psychiatry. 2022 Apr 4;12:139. doi: 10.1038/s41398-022-01903-3 (PMC8980007; doi:10.1038/s41398-022-01903-3)
Supplement: Supplementary file 1 — Supplementary figure legends [file 41398_2022_1903_MOESM1_ESM.docx]

**Figure S1. Relationship between desmosterol and 7DHC concentrations in cerebellum and PFC.** (A) Desmosterol, (B) 7-dehydrocholesterol concentrations (ng/mg tissue) was compared between cerebellum (CBL) and pre-frontal cortex (PFC) of each individual. Control (CTRL) and depressed (DEP) subjects are indicated with red and blue dots, respectively.

**Figure S2. Posterior probability distribution of the difference in mean desmosterol concentrations between control and depressed groups.**  The concentrations in ng/mg tissue is plotted for (A) pre-frontal cortex (PFC) and (B) cerebellum (CBL). Dark horizontal line above the axis indicates the 95% Highest Density Interval. Green dotted line indicates the probability that the difference in the means is less than or equal to zero.

**Figure S3. Posterior probability distribution of the difference in mean 7-dehydrocholesterol** **concentrations between control and depressed groups.**  The concentrations in ng/mg tissue is plotted for (A) pre-frontal cortex (PFC) and (B) cerebellum (CBL). Dark horizontal line above the axis indicates the 95% Highest Density Interval. Green dotted line indicates the probability that the difference in the means is less than or equal to zero.

**Figure S4. 24S-hydroxycholesterol concentrations in CBL and PFC.** Concentrations are in ng per mg tissue.
