# Supplementary figures and images for: Desmosterol and 7-dehydrocholesterol concentrations in post mortem brains of depressed people: The role of trazodone
Source: Transl Psychiatry. 2022 Apr 4;12:139. doi: 10.1038/s41398-022-01903-3 (PMC8980007; doi:10.1038/s41398-022-01903-3)

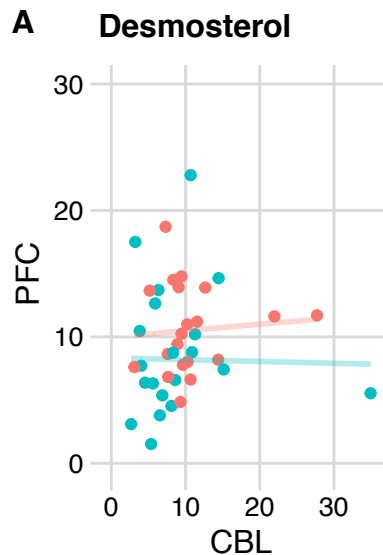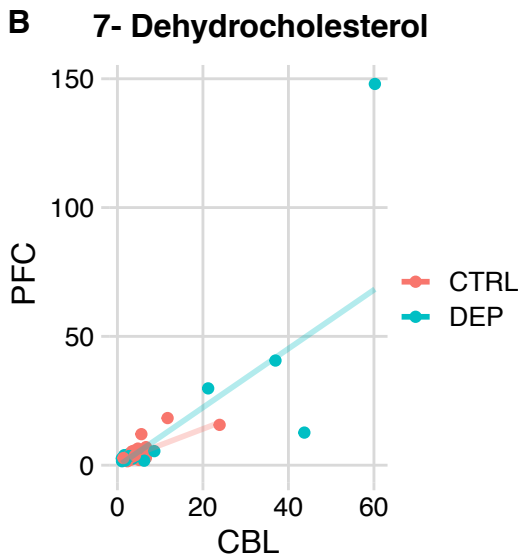

Supplement: Supplementary file 2 — Figure S1 [file 41398_2022_1903_MOESM2_ESM.pdf]

**A****PFC**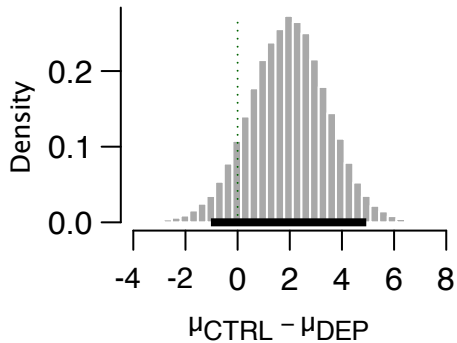**B****CBL**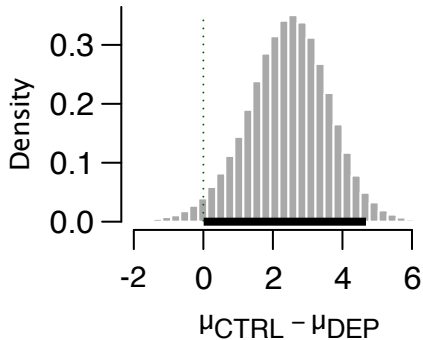

Supplement: Supplementary file 3 — Figure S2 [file 41398_2022_1903_MOESM3_ESM.pdf]

**A**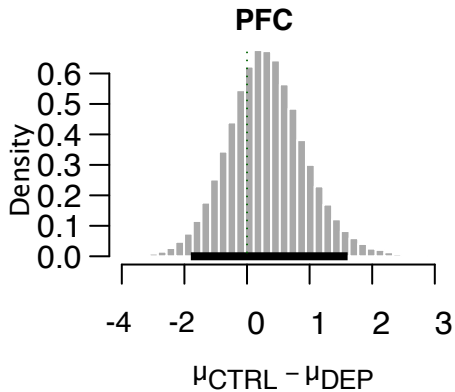**B**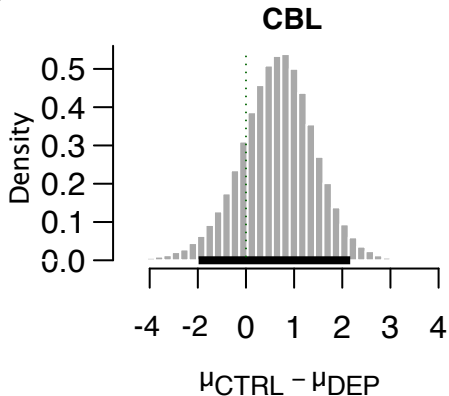

Supplement: Supplementary file 4 — Figure S3 [file 41398_2022_1903_MOESM4_ESM.pdf]

24S-hydroxycholesterol

50  
40  
30  
20  
10  
0

CBL

PFC

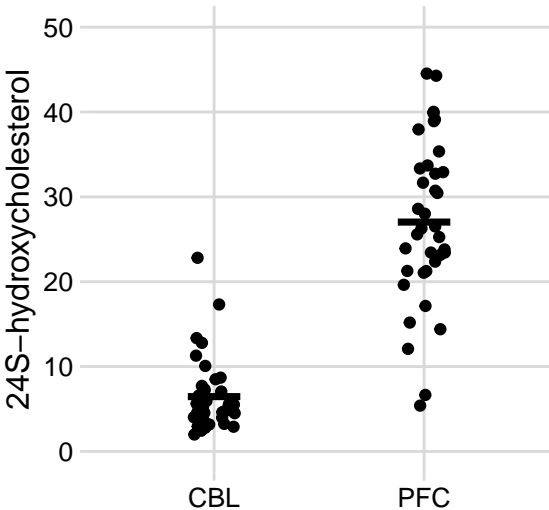

Supplement: Supplementary file 5 — Figure S4 [file 41398_2022_1903_MOESM5_ESM.pdf]
